# Editorial: Everything edamame: biology, production, nutrition, sensory and economics, volume II

**DOI:** 10.3389/fpls.2024.1488772

**Published:** 2024-09-18

**Authors:** M. Luciana Rosso, Bo Zhang, Martin M. Williams, Xujun Fu, Jeremy Ross

**Affiliations:** ^1^ School of Plant and Environmental Sciences, Virginia Polytechnic Institute and State University, Blacksburg, VA, United States; ^2^ Global Change and Photosynthesis Research Unit, USDA-Agricultural Research Service, Urbana, IL, United States; ^3^ Institute of Crop and Nuclear Technology Utilization, Zhejiang Academy of Agricultural Sciences, Hangzhou, China; ^4^ Crop, Soil, and Environmental Sciences, University of Arkansas System Division of Agriculture, Little Rock, AR, United States

**Keywords:** edamame, vegetable soybean, GWAS, GS, CSSLs, sensory, seedling emergence

Edamame, or vegetable soybean, is a valuable source of protein, soluble sugars, starch, dietary fiber, minerals, isoflavones, and vitamins. Traditionally cultivated in East Asia, edamame has gained popularity worldwide due to its rich nutritional profile and consumer appeal, especially in North America. The increasing demand for plant-based proteins has positioned edamame as a key component in sustainable diets, with ongoing efforts to enhance its sweetness and nutritional content to meet consumer preferences. This editorial introduces the second volume in the series “Everything Edamame: Biology, Production, Nutrition, Sensory and Economics” bringing together a collection of seven articles that contribute to a deeper understanding of edamame and related legumes. These studies span a wide range of Research Topics, including genetic diversity, genomic selection, phenotypic analysis, texture evaluation, and environmental factors influencing seedling establishment. Additionally, they explore the role of soluble sugar content, protein, sucrose, alanine, and oil content, as well as seed-pod characteristics, and the impact of processing on legume quality. All these efforts are directed toward enhancing the production, quality, and marketability of edamame, aiming to meet consumer demand while supporting sustainable agricultural practices.

One of the keystone studies in this volume introduces a groundbreaking approach to enhancing genetic diversity in vegetable soybeans by employing a multiple imputation (MI) method to address the issue of missing phenotype data (Huang et al.). By imputing missing phenotypic data across diverse genotypes, this method allows for a more accurate and comprehensive assessment of genetic diversity in core collections. The study found that the MI-based core collection retained high diversity and represented the entire collection more effectively than previous methods. This research is highly impactful for the vegetable soybean industry as it provides a robust tool for plant breeders to better utilize genetic resources, leading to the development of improved soybean varieties with greater genetic diversity. This advancement is crucial for ensuring the sustainability and adaptability of vegetable soybeans in various environmental conditions, ultimately enhancing their production and marketability globally.

Building on the theme of genetic improvement, Zheng et al. explore the construction of chromosome segment substitution lines (CSSLs) and the inheritance patterns of seed-pod characteristics in wild soybeans. This study is particularly significant for edamame breeding, as it bridges the gap between wild and cultivated soybeans, offering valuable insights into how beneficial traits from wild relatives—such as disease resistance, drought tolerance, and enhanced seed quality—can be introduced into edamame varieties. By mapping these traits to specific chromosome segments, the research provides a clear roadmap for breeding programs aimed at developing new edamame varieties that can not only thrive in challenging environments but also meet the high standards of flavor, texture, and nutritional quality demanded by consumers.

The exploration of genetic markers is further advanced by two genome-wide association studies (GWAS) that focus on identifying loci associated with key nutritional traits in edamame. Wang et al. investigate the genetic basis of sucrose and alanine content in edamame beans, identifying 45 single nucleotide polymorphisms (SNPs) associated with sucrose content and 25 SNPs linked to alanine levels. They highlight key candidate genes such as *Glyma.18G193600*, which encodes a fructose-1,6-bisphosphatase involved in the sucrose synthesis pathway, and *Glyma.17G070500*, associated with alanine biosynthesis. Wang et al. also identified three Plant Introduction (PI) accessions (PI 532469, PI 243551, and PI 407748​) with high sucrose and high Ala content that could be used for the breeding of sweeter edamame varieties. Meanwhile, Lu et al. focus on soluble sugar content, identifying *Glyma.02G294000* as a crucial gene influencing this trait. This gene is part of the O-Glycosyl hydrolases family, which is involved in starch and sucrose metabolism, affecting the degradation of Uridine diphosphate glucose (UDP-glucose) and subsequently influencing the soluble sugar content. Their study also identified *Glyma.02G293900*, which is involved in carbohydrate metabolism, and *Glyma.02G294900*, both of which were positively correlated with soluble sugar content. The findings from Lu et al. provide a comprehensive understanding of the genetic factors regulating soluble sugar content in soybean, with *Glyma.02G294000* showing a significant negative correlation with sugar levels, making it a key target for breeding programs aimed at improving the sweetness of edamame. These studies are crucial as they link specific genetic regions to traits that directly affect the flavor and nutritional profile of edamame, key factors in consumer preference and market success. By identifying genetic loci, they pave the way for marker-assisted breeding, enabling faster development of varieties that meet consumer demands for taste and nutrition.

In the context of genomic selection (GS), Sun et al. conducted a comprehensive study to improve protein and oil content in soybean seeds. Utilizing the Ridge Regression Best Linear Unbiased Predictor (RR-BLUP) model and analyzing 1,007 soybean accessions, the study found that GS can achieve high prediction accuracy, particularly for oil content, which was found to be higher than that for protein content. The study also highlighted that the size and genetic diversity of the training population significantly affect prediction accuracy, with larger and more genetically diverse populations yielding better results. Additionally, the inclusion of approximately 3,000 markers strongly associated with the targeted traits was shown to improve the prediction accuracy, making GS a promising tool for accelerating the development of soybean varieties with enhanced nutritional profiles. This GS approach is particularly beneficial to the edamame industry, enabling faster and more efficient production of high-protein, high-oil varieties that meet consumer demands for healthier food options.

While GS drives improvements in the nutritional quality of soybeans, sensory qualities of legume products, particularly texture, are equally critical to consumer acceptance and marketability. Miller et al. conducted a comparative analysis of the texture of edamame, peas, and lima beans using advanced texture analysis techniques. Texture, an often-overlooked aspect of food quality, plays a significant role in consumer satisfaction, especially as the demand for plant-based proteins grows globally. The study reveals that edamame, with its unique textural properties, stands out among legumes, making it an attractive option for a variety of culinary applications. The research emphasizes the importance of standardized methods in texture analysis to ensure consistent product quality across different batches and processing methods. Such standardization is crucial for maintaining consumer trust and expanding the market for edamame-based products.

Building on the importance of quality from seed to plate, Li et al. extend the discussion to the early stages of plant development by investigating how seed physiological traits and environmental factors influence seedling establishment in edamame. This study provides practical insights into the conditions necessary for optimal germination and seedling growth, which are critical for achieving high yields in edamame cultivation. They recommend maintaining soil temperatures between 25°C and 33°C to achieve over 90% emergence within 3-3.5 days and highlight the importance of proper soil moisture management, especially for larger seeds. The study also suggests using fungicides or biological agents, like *Trichoderma harzianum*, to protect against soilborne pathogens, particularly in poorly drained soils. These strategies are vital for improving germination and ensuring successful edamame establishment, especially under the unpredictable conditions of climate change.

Together, these articles collectively deepen our understanding of the genetic, phenotypic, and environmental factors influencing edamame quality and productivity. These studies shared a focus on enhancing the quality and resilience of edamame. The genetic diversity ensured through advanced phenotype imputation methods (Huang et al.) provides a rich foundation for the genomic selection processes discussed by Sun et al. Similarly, the insights gained from studying the inheritance of seed-pod traits in wild soybean (Zheng et al.) can inform breeding programs aimed at improving traits identified through GWAS (Wang et al.; Lu et al.). Moreover, the emphasis on sensory qualities, as explored by Miller et al., ties directly into the consumer acceptance that ultimately drives market success, which is dependent on the nutritional and textural qualities discussed throughout this volume. As the challenges of food security and climate change intensify, the Research Topics in this collection are crucial for developing superior vegetable soybean varieties, ensuring edamame’s role in sustainable agriculture and global nutrition. Integrating these insights into breeding strategies is key to realizing edamame’s full market potential.

